# A8 MICROBIAL ACTIVATION OF INTESTINAL DENDRITIC CELLS IS CRITICAL FOR THE ESTABLISHMENT OF NORMAL BEHAVIOR

**DOI:** 10.1093/jcag/gwac036.008

**Published:** 2023-03-07

**Authors:** N Kraimi, V Philip, H Zhang, J Lu, G De Palma, E F Verdu, K D McCoy, S Hapfelmeier, A J Macpherson, F Chirdo, M Surette, F Liu, S M Collins, P Bercik

**Affiliations:** 1 Medicine, Farncombe Family Digestive Health Research Institute, McMaster University, Hamilton; 2 Campbell Family Mental Health Research Institute, the Centre for Addiction and Mental Health, Toronto, Canada; 3 Department of Biomedical Research, University Hospital, Bern, Switzerland; 4 Physiology and Pharmacology, Snyder Institute, Cumming School of Medicine, University of Calgary, Calgary, Canada; 5 University of Bern, Institute for Infectious Diseases, Bern, Switzerland; 6 Instituto de Estudios Inmunologicos y Fisiopatologicos - IIFP (UNLP-CONICET), La Plata, Argentina

## Abstract

**Background:**

Accumulating evidence suggests that gut microbiota affects brain development and its function. It is well known that compared with conventional mice (SPF), germ-free (GF) mice display higher exploratory behavior, which normalizes after bacterial colonization. However, little is known about the underlying mechanisms and first critical steps initiating microbiota-gut-brain communication, which lead to establishment of normal behavior.

**Purpose:**

To investigate the role of immune system in the establishment of normal behavior after bacterial colonization.

**Method:**

We assessed behavior in GF mice before and after colonization with SPF microbiota, Altered Schaedler Flora (ASF) or the single bacterial strain *E. coli* JM83, and compared them to SPF mice, using the light-dark preference and tail suspension tests. Levels of brain-derived neurotrophic factor (BDNF) and c-Fos expression were measured by immunofluorescence in the hippocampus and amygdala. Colonic and brain gene expression were assessed using a NanoString technology. The immunodeficient MyD88^-/-^ Ticam1^-^ and SCID mice were used to study the role of the innate and adaptive immune systems. To demonstrate the role of the dendritic cells (DCs), we measured behavior before and after mono-colonization with *E. coli* JM83 in GF mice treated with cosalane and fingolimod, that inhibit DCs activation and migration, respectively. Brain levels of CD11b, CD11c and CD103 as DCs markers was assessed by immunofluorescence.

**Result(s):**

Compared to SPF mice, GF mice showed higher exploratory and less depressive-like behavior. The ex-germ-free mice colonized with ASF microbiota, or mono-colonized with *E. coli* JM83 showed similar normalization of behavior as those colonized with SPF microbiota. Mono-colonization with *E. coli* reduced both BDNF and c-fos levels in the hippocampus and amygdala. While colonization of GF SCID mice induced same change in behavior as in wild-type mice, GF MyD88^-/-^Ticam1^-/-^ mice did not alter their behavior. Mono-colonization affected multiple genes in the colon and the brain, associated with innate immunity and neural plasticity. Treatment with both cosalane and fingolimod prevented behavioral changes after colonization, which was paralleled by absence of CD11b^+^CD103^+^CD11c^+^ cells in the brain, otherwise found in high numbers in control mono-colonized mice and absent in germ-free mice.

**Conclusion(s):**

The innate immune system, through activation and migration of intestinal dendritic cells into the brain, initiates the neuro-immune signaling within the gut-brain axis and leads to normalization of behavior after bacterial colonization. Our findings may impact several psychiatric conditions, in which altered innate immune signaling has been implicated.

**Please acknowledge all funding agencies by checking the applicable boxes below:**

CIHR, Other

**Please indicate your source of funding;:**

Balsam Family Foundation

**Disclosure of Interest:**

None Declared

